# LEARN 2 MOVE 2-3: a randomized controlled trial on the efficacy of child-focused intervention and context-focused intervention in preschool children with cerebral palsy

**DOI:** 10.1186/1471-2431-10-80

**Published:** 2010-11-08

**Authors:** Marjolijn Ketelaar, Anne JA Kruijsen, Olaf Verschuren, Marian J Jongmans, Jan Willem Gorter, Johannes Verheijden, Heleen A Reinders-Messelink, Eline Lindeman

**Affiliations:** 1 Rehabilitation Center De Hoogstraat, Center of Excellence for Rehabilitation Medicine Utrecht, Utrecht, the Netherlands; 2 University Medical Center Utrecht, Rudolf Magnus Institute of Neuroscience, Department of Rehabilitation, Nursing Science and Sports, Utrecht, the Netherlands; 3 Utrecht University, Faculty of Social Sciences, Department of Pedagogical and Educational Sciences, Utrecht, the Netherlands; 4 University Medical Center Utrecht, Wilhelmina Children's Hospital, Department of Pediatric Psychology, Utrecht, the Netherlands; 5 CanChild Centre for Childhood Disability Research, McMaster University, Hamilton, Ontario, Canada; 6 BOSK, Association of physical disabled persons and their parents, Utrecht, the Netherlands; 7 Partner of NetChild, Network for Childhood Disability Research in the Netherlands, the Netherlands; 8 Rehabilitation Center 'Revalidatie Friesland', Beetsterzwaag, the Netherlands; 9 Center for Rehabilitation, University Medical Center Groningen, Groningen, the Netherlands

## Abstract

**Background:**

Little is known about the efficacy and the working mechanisms of physical and occupational therapy interventions for children with cerebral palsy (CP). In recent years a shift from a child-focused intervention approach to a more context-focused intervention approach can be recognized. Until now the evidence on the efficacy and the working mechanisms of these interventions for children with CP is inconclusive. This study aims to evaluate the efficacy and working mechanisms of two intervention approaches compared to regular care intervention in improving mobility and self-care skills of children (2-3 years) with CP and their families: a child-focused intervention approach and a context-focused intervention approach.

**Methods/Design:**

A multi-centre, randomized controlled trial research design will be used. Ninety-four children with CP (Gross Motor Function Classification System (GMFCS) level I-IV; age 2 to 3 years), their parents, and service providers (physical and occupational therapists) will be included. During a period of six months children will receive child-focused, context-focused or regular care intervention. Therapists will be randomly assigned to deliver either a child-focused intervention approach, a context-focused intervention approach or regular care intervention. Children follow their therapist into the allocated intervention arm. After the six months study-intervention period, all participants return to regular care intervention. Outcomes will be evaluated at baseline, after six months and at a three months follow-up period. Primary outcome is the capability of functional skills in self-care and mobility, using the Functional Skills Scale of the Pediatric Evaluation of Disability Inventory (PEDI). Other outcomes will be quality of life and the domains of the International Classification of Functioning, Disability and Health - for Children and Youth (ICF-CY), including body function and structure, activities (gross motor capacity and performance of daily activities), social participation, environmental variables (family functioning, parental empowerment).

**Discussion:**

This paper presents the background information, design, description of interventions and protocol for this study on the efficacy and working mechanisms of child-focused intervention approach and context-focused intervention approach compared to regular care intervention in mobility and self-care skills of children (2-3 years) with CP.

**Trial registration:**

This study is registered in the Dutch Trial Register as NTR1900

## Background

The present paper describes the background and design of LEARN 2 MOVE 2-3 (L2M 2-3). This project is part of the Dutch national LEARN 2 MOVE research program, which evaluates interventions in rehabilitation for children and adolescents with Cerebral Palsy (CP) in different age cohorts [1-3].

For children with CP at young age in general a shift from a child-focused intervention approach to a more context-focused intervention approach can be recognized. Until now however, the evidence on the efficacy and the working mechanisms of these intervention approaches for children with CP are inconclusive. Moreover, it is not known which interventions are being used in clinical practice, and how these developments transfer into clinical practice. L2M 2-3 focuses on these questions.

**Figure 1:**
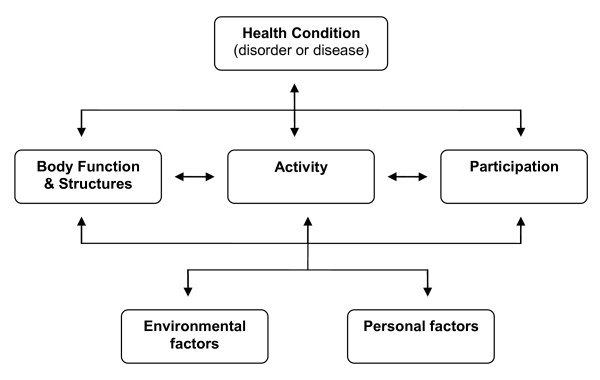
**ICF-model**.

The child-focused intervention approach and context-focused intervention approaches are based on different principles as can be described in terms of the International Classification of Functioning, Disability and Health (ICF;[4]) and the recently published derived version of it, the International Classification of Functioning, Disability and Health for Children and Youth (ICF-CY;[5]). In the ICF and ICF-CY a persons functioning can be described on various levels (Figure 1) in its relation with environmental and personal factors.

Both the child-focused intervention approach and the context-focused intervention approach aim to improve activities and participation of the children. They differ however in the assumed pathways how to reach the goals at the level of activities and participation.

The child-focused intervention approach focuses primarily on remediation of *body function and structure *as the starting point of intervention. Interventions based on such an approach include: facilitation of normal movement patterns and postural control through physical handling [6-8], maintaining range of motion and joint alignment through stretching, casting and splinting [9,10], strength training [11-13], and treadmill training [14]. These strategies, focusing on remediation of body function and structure, can be performed in isolation or as part of practicing activities.

The context-focused intervention approach focuses primarily on changing constraints in the environment or task as the starting point of intervention. Both the physical environment and the social environment of the child are emphasized [15]. In the context-focused intervention approach the therapist will not elicit specific responses by handling the child, but will provide an environment that enables the child to learn to perform self-initiated actions within naturally occurring restraints. The child can then become an active problem solver throughout the day instead of being a passive recipient of treatment. The social context of the child, especially the family, plays an important role.

Currently, *CanChild *Centre for Childhood Disability Research in Canada is performing a study into the efficacy of a child-focused intervention approach compared to a context-focused intervention approach, the Focus on Function Study [16]. L2M 2-3 builds on the Focus on Function study in the way that the efficacy of the same child-focused intervention approach and context-focused intervention approach will be compared with the same outcome measures in different countries, and by adding the comparison with regular care. Little is known about the actual content of current interventions for young children with CP. They probably consist of a mix between the child-focused intervention approach and the context-focused intervention approach, without a clear theoretical base. To provide children with CP with the best intervention, it is necessary to understand which interventions are effective and why these interventions are effective.

The aim of the present project is to evaluate the efficacy of a child-focused intervention approach or a context-focused intervention approach versus regular care intervention in improving daily functioning of children (2-3 years) with CP and their families. In defining daily functioning it is important to distinguish three constructs of activity: capacity, capability, and performance. Capacity describes what a person can do in a standardized, controlled environment. Capability describes what a person can do in his/her daily environment. Performance describes what a person actually does do in his/her daily environment [17,18].

Primary outcome in the present study is the capability of functional skills in self-care and mobility, as reflected in the first research question. Other domains and concepts of the ICF-CY are secondary outcomes, leading to the following research questions. Which of the three intervention approaches results in:

i. greater achievement of capability in functional skills in mobility and self-care of the children?

ii. greater independence in the performance of mobility and self-care of the children?

iii. greater achievement of capacity of motor skills of the children in a standardized environment?

iv. greater participation of children and their families?

v. better quality of life of the children and their families?

In addition to the questions on efficacy, the working mechanisms of the child-focused intervention approach and the context-focused intervention approach will be examined. Parental stress, empowerment and family-centeredness of care will be analyzed in the three intervention groups. Moreover, variables that might be related to the efficacy of the intervention approaches and that might explain possible variability between children will be examined. In a longitudinal study of our research group on the development and determinants of daily activities in children with CP named PERRIN (Pediatric Rehabilitation Research in the Netherlands), and from recent literature, insight has been gained in child- and family-variables that possibly explain more about the working mechanisms of the interventions. These variables are: gross motor abilities [19,20], manual abilities [21,22], cognition of the child [21,23,24], and coping style of the parents [25-27]. We will collect data on these variables to get more insight in possible variability between children. Moreover, to get more insight into the working-mechanisms of the intervention approaches, part of the parents will be interviewed on their experiences with the interventions and therapists will be asked about preferences for intervention.

## Methods/Design

### Design

A multi-centre, randomized controlled trial research design will be used. The study protocol is approved by the Medical Ethics Committee of the University Medical Centre Utrecht, the Netherlands.

### Setting

The study is a multi centre study and will be conducted in approximately 15 rehabilitation centers in the Netherlands between September 2009 to December 2011.

### Study population

Ninety-four children with CP will be included. The inclusion criteria are:

- Children with diagnosis of CP [28];

- Children classified in Levels I-IV at the Gross Motor Function Classification System (GMFCS; [29,30]);

- Children enrolled in pediatric rehabilitation care, with at least physical or occupational therapy;

- Children receiving therapy with a frequency of at least three sessions per month;

- Children aged 24 months to 47 months (2-3 years) at the time of recruitment.

The exclusion criteria are:

- Children with planned surgery or medical changes during the study that may affect their motor function;

- Children whose families feel uncomfortable or unable to respond to interviews and questionnaires in Dutch (the language of all the study materials);

- Since this an efficacy study, parents who state that they will not be able to adhere to one or two of the intervention approaches or to the treatment schedule, will not be entered in the study.

Children and their parents will be approached by their treating pediatric physician. They will receive an information letter, an informed consent form, a brochure about the LEARN 2 MOVE 2-3 study, and a brochure about participating in scientific research. The researcher will contact the parents after two weeks to respond to possible queries and provide additional information when necessary. Children of parents who give informed consent will participate in the study.

### Sample size

Sample size was calculated for the Pediatric Evaluation of Disability Inventory (PEDI) (based on data from an earlier study [31]; with a subgroup analysis of children of 2-3 years) using the following assumptions in a one-way analysis of variance power analysis (based on a power of 80% and a two-sided α-value of 0.05). An estimated difference in the change scores between the context-focused intervention approach group and the regular care intervention group of 4.5 points (SD = 6.0) on the PEDI. Sigma for the three groups = 2.1. Taking into account the clustered nature of the sample of participating children a correction will be used, based on an average cluster size (number of children per therapist) of 2, and an ICC of 0.1. These specifications lead to a required total sample size of 85. Expecting a 10% drop-out in total 94 children will be recruited for the present study.

### Study procedures

The three interventions arms will be distributed within a centre. The physical and occupational therapists who agreed to participate will be randomized in one of the three intervention arms by block randomization, with a block size of six. Children will follow their therapist into the intervention group that the therapist was assigned to. The therapist who provides the study intervention to the child will be the prime therapist for the children during the six months study-intervention period. Furthermore, the prime therapist is the one who will document on the session documentation, will set goals with parents and is the contact person for the researchers. The other therapists who treat the child will be available for expert information consultation. Therapists can participate in the study with a maximum of two children at the same time and with a maximum of four children overall in the study. We expect an average of two children per therapist.

**Figure 2:**
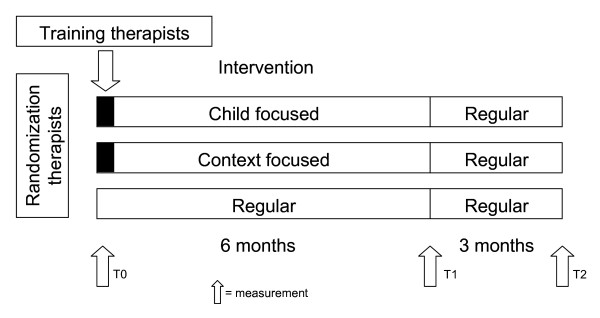
**Design LEARN 2 MOVE 2-3 study**.

The study procedure for the participants is represented in Figure 2. After randomization procedures, therapists in the child-focused intervention approach and therapists in the context-focused intervention approach group will receive additional training. This training will start with both groups together (introduction and background of the study, and steps in discussing and formulating problems and goals with parents using the Canadian Occupational Performance Measure (COPM;[32]) and Goal Attainment Scaling (GAS;[33]). The second part of the training will be completed separately for each group and focuses on implementing the specific components of child-focused intervention approach or context-focused intervention approaches. Expert consultation will be available for therapists in each intervention group. The researchers and the consultant for each intervention will conduct the training sessions, providing both theoretical and applied information about the assessment and intervention protocols. Therapists will discuss case scenarios, and view videos of children. The training and training syllabus are developed based on materials from the Focus on Function study of *CanChild*. During the study therapists in the child-focused intervention approach and context-focused intervention approach groups will document sessions on standardized forms, and in the stage of goal-setting, will make a videotape of the child to make clear what the goals of the individual child and the parents are. The therapists will receive feedback on the session documentation and the video-tape which they send to the researchers. The therapists will have access to a consultant and the researchers, for support, problem solving, and resources. Therapists in the regular care intervention arm will be asked to work the way they are used to do, and at the end of the study their session documentation will be collected.

After inclusion in the study a baseline measurement (T0) will take place. Children will be assessed and parents will fill out questionnaires and will be interviewed on the measures as described in the Section Study Parameters. Parents can choose whether they would like to fill out the questionnaires on a digital or a paper version. Thereafter participants will follow their therapist into one of the three intervention groups for a six months study-intervention period. After these six months the second measurement (T1) will take place, after which all participants return to regular care intervention. At a three months follow-up period the third measurement (T2) will take place. Independent assessors who are blind for intervention allocation will asses the children and their parents.

### Interventions

Children included in the study typically receive physiotherapy and/or occupational therapy with a frequency of at least three times a month. During the six months study-intervention period, all children will receive at least 18 sessions of intervention. The amount of intervention sessions will be documented in the session documentation of the therapists.

In the child-focused intervention approach and context-focused intervention approach the procedure of problem definition, priorities, goals, analysis, sub goals and evaluation will be the same as much as possible, to avoid a confounding effect by procedure (see Table 1).

**Table 1:** Comparison of procedure of the child-focused, context-focused and regular care intervention

	Child-focused	Context-focused	Regular care
**Problem definition**	COPM	COPM	No instructions on documentation, therapists will document the way they are used to do. Documentation will be collected after the end of the study.
	
**Priorities**	COPM	COPM	
	
**Goals**	- Goal identification according to the COPM - Goals formulated SMART^1^	- Goal identification according to the COPM, with parents - Goals formulated SMART^1^	
	
**Analysis**	Arrange constraints and enablers in ICF-CY - focus on constraints and facilitators in the child	Arrange constraints and enablers in ICF-CY - focus on constraints and facilitators in the environment and the task	
	
**Sub-goals**	Sub-goals formulated SMART^1^	Sub-goals formulated SMART^1^, with parents	
	
**Intervention**	Focus on remediation of the child's abilities through changing the components of body function and structure	Focus on functional performance trough changing constraints in the task and/or the environment	
	
**Evaluation**	Therapist evaluate goal, when attained a new goal will be defined	Therapist evaluate with parents goal, when attained a new goal will be defined with parents	

^1^Specific Measurable Attainable Realistic Timely

To ensure that the procedures in the child-focused intervention approach and the context-focused intervention approach are the same, forms will be used to guide the steps.

#### Child-focused intervention

The child-focused intervention approach focuses primarily on remediation of *body function and structure *as the starting point of intervention. The task analysis focuses on constraints and facilitators in the child. Therapeutic techniques will be used to improve the child's body functions and structure, such as strength, coordination, balance, quality of movement, and range of motion. Strategies, focusing on remediation of body function and structure, can be performed in isolation or as part of practicing activities. The therapist is the primary decision maker in the intervention.

In the training therapists will learn child-focused intervention principles. Therapists will be instructed to achieve the goals primarily through changing the components of body function and structure within the child.

#### Context-focused intervention

The context-focused intervention approach focuses primarily on changing constraints in the environment or task as the starting point of intervention. The task analysis focuses on constraints and facilitators in the environment and the task. For young children with CP (2-3 years) the most important contextual factors are situated in the home environment. In this approach therefore, the home environment is taken into consideration [34], and there is a strong collaboration between therapists and parents recognizing parents' role as advocates and decision makers for their child [35,36]. In this approach, the task of the therapist is not to elicit specific responses by handling the child, but to provide an environment which enables the child to learn to perform self-initiated actions within naturally occurring restraints.

In the training the therapists will learn the context-focused intervention principles. Therapists will be instructed to achieve the goals primarily through changing the environment and tasks. Therefore, the therapists will challenge the children with frequent and varied practice within the context of daily activities and routines.

#### Regular care

The regular care intervention in this study will be like the intervention the children received before participating in the study and continues throughout the study period. Regular care consists probably of a mixture of child-focused intervention approaches and context-focused intervention approaches, with the balance between child-focused and context-focused approaches based on the individual therapist's view.

### Study parameters

#### Primary study outcome

The primary outcome of the study will be the capability of functional skills in mobility and self-care using the Functional Skills Scale of the PEDI *(PEDI-FSS*;[37]). The PEDI is a standardized assessment instrument using parental reports through structured interview, and consists of the Functional Skills Scale (FSS), and the Caregiver Assistance Scale (CAS), both consisting of three domains: self-care, mobility, and social function. For the purpose of the present study the self-care and mobility domain will be used. The PEDI-FSS assesses what a child can do in his/her daily environment. Each question is scored as positive (score 1) or negative (score 0). A positive score is given when the child is capable of accomplishing the activity. The Dutch adaptation and translation of the PEDI, the PEDI-NL[38], which has good psychometric properties, will be used [39].

#### Secondary study outcomes

Performance of mobility-related activities and self-care-related activities will be measured by the *Caregiver Assistance Scale of the PEDI (PEDI-CAS*;[37]). This scale measures the level of caregiver assistance a child receives to accomplish the activities. It concerns the child's actual behavior in his or her daily environment.

To set intervention goals and to measure the amount of successfully reached goals after the six months study-intervention period the *COPM *will be used [32]. The COPM is an individualized measure designed to detect problems in various areas of daily functioning and to assess changes in self-perceived performance and satisfaction over time through a semi-structured interview. The COPM has robust psychometric properties for use in a pediatric clinical trial [40]. The Dutch version of the COPM has shown good validity [41,42].

Capacity of motor skills will be assessed with the *Gross Motor Function Measure (GMFM*;[43]). The GMFM is a standardized observational instrument that has been developed to measure the gross motor function in children with CP in a specific test situation, without the use of mobility aids or orthosis. The original GMFM consists of 88 items grouped into five dimensions of gross motor function: lying and rolling; sitting; crawling and kneeling; standing; and walking, running, and jumping. There is also a 66-item version of the GMFM available, in which 22 items of the GMFM-88 are removed, many of them in the dimension of lying and rolling. The most recent development with the GMFM is the GMFM-66-IS (Item-Set) in which four items sets are used to determine which items need to be examined. This abbreviated version of the GMFM is less time consuming and has shown to be valid en reliable [44]. In the present study, with a study population of children with CP aged 2 to 3 years, we expect that the lying and rolling and sitting dimensions can provide essential information. Therefore all items of these two dimensions of the GMFM-88 will be assessed. In addition the GMFM-66-IS will be used to determine which items on the other dimensions need to be examined for each individual child. The Dutch translation of the GMFM-88, which has good psychometric properties comparable to those of the original GMFM will be used [45,46].

Participation of the child in daily life activities will be measured by the *Preschool Children's Assessment of Participation and Enjoyment (Pre-School CAPE)*. The Pre-school CAPE is a measure based on the CAPE (Children's Assessment of Participation and Enjoyment) [47]. The CAPE is a self-reported 55-item questionnaire to measure participation and enjoyment in children 6 to 21 years of age. The CAPE has shown to be sensitive in measuring change in participation during intervention [48] and shows good psychometric properties [35,49]. The CAPE was adapted for children at a younger age to the Pre-school CAPE by changing some items and parents will fill in the questionnaire for their children. This questionnaire consists of 48 items to measure the participation in everyday activities in children over the past four months. The Pre-school CAPE measures on five domains: play activities, skill development, active physical recreation, social activities and other activities. The questionnaire provides information on the diversity and intensity of these activities.

Parental perception of the participation of the family will be measured by the *Family participation questionnaire*. In a semi-structured interview parents are asked three questions on daily activities of the family, and on family participation. The questionnaire has shown shows good inter-rater reliability in the PERRIN CP 0-5 study[50].

Quality of life of the child will be measured by *TNO-AZL Preschool Children Quality of Life Questionnaire (TAPQOL*;[51]). The TAPQOL is a 43-item questionnaire to measure parents' perception of the health-related quality of life in preschool children. Parents fill in the questionnaire as a proxy for their child. The questionnaire consists of 12 scales on the domain of physical, social, cognitive and emotional functioning. The occurrence of these items is assessed and at some items also the wellbeing of the child in relation to the item is scored. The TAPQOL showed to have a good reliability [51,52].

Quality of life of the parents will be measured by three questions based on a national survey of 'Statistics Netherlands', the *Questions Quality of Life*. The answers of these questions can be compared with Dutch norms.

Parental stress in relation to raising their child will be measured by the *Nijmeegse Ouderlijke Stress Index - Kort (NOSI-K*;[53]). The NOSI-K is with 25 items the short version of the NOSI, which is a Dutch adaptation of the Parenting Stress Index (PSI). The NOSI-K has shown good psychometric properties [53].

The empowerment of the family will be measured by the *Family Empowerment Scale (FES*;[54]). The FES is a 34-item questionnaire to measure empowerment in families of children with emotional, behavioral or mental disorders. The FES consists of three domains about family, child's services, and parents' involvement in the community. The FES has good psychometric properties [54,55].

The family-centeredness of care as rated by the parents will be measured by the *Measure of Processes of Care (MPOC*;[56]). The MPOC is a 56-item questionnaire to assess the extent to which parents perceived the family centeredness of the health care their child received in the past year. The questionnaire consists of five scales; enabling and partnership, providing general information, providing specific information about the child, coordinated and comprehensive care for the child and family, and respectful and supportive care. The occurring of these items will be assessed as well as how important they are to parents. In this study the Dutch version of the MPOC will be used, with good psychometric properties [57].

The family-centeredness of care as rated by professionals will be measured by the *Measure of Processes of Care for service providers (MPOC-SP*;[58]). The MPOC-SP is a 27-item questionnaire to assess to which extent the care that service providers provided the past year was family-centered. The questionnaire consists of four scales. In this study the Dutch version of the MPOC-SP will be used, with good psychometric properties [58,59].

#### Other study parameters

Based on the literature, it is assumed that some child- and family variables might be important in explaining effects in different groups. Therefore, the following variables will be registered systematically, and will be analyzed as potential determinants:

The gross motor ability level of the child will be classified by the *GMFCS *[29,30]. The GMFCS is a classification system based on the self-initiated movements of the child. The system consists of a 5-level ordinal scale, which describes differences in severity of mobility abilities. Level I represents the best gross motor function, level V the least. The GMFCS shows good psychometric properties [29,30].

Manual skills will be classified by the *Manual Ability Classification System (MACS*; [60]). The classification system consists of five levels, from level I 'Handles objects easily and successfully' to level V 'Does not handle objects and has severely limited ability to perform even simple actions'. The MACS has shown good reliability in children 2-5 years of age [61]. Validity of the MACS was shown in children 4-18 years of age [60]. The validity in children 2-5 years of age has not been studied yet.

Type of CP will be classified according to *Surveillance of Cerebral Palsy in Europe (SCPE) - guideline *[62]. This guideline classifies CP in the subtypes of spastic, dyskinetic, or ataxic. Also the topographical distribution is classified in children with spastic CP; unilateral involvement (hemiplegia) versus bilateral involvement (diplegia, tetraplegia).

Cognitive functioning of the child will be measured by the *Bayley Scales of Infant Development-Second edition (BSID-II) - Mental Scale*, with good psychometric properties [63].

Coping style of the parents will be measured by the *Utrechtse Copinglijst (UCL)*, a Dutch 47-item questionnaire to determine how parents handle problems and events in daily life, with sufficient psychometric properties [64].

*Session documentation *of the therapists will be collected. Therapists in the child-focused intervention approach and in the context-focused intervention approach group will document on special forms and will send in their documentation during the intervention period to control the therapist's adherence. Experience from previous work [31,65,66] has shown that therapists are able to implement treatment protocols in a reliable and valid manner. Therapists in the regular care intervention group will not receive instructions on documentation and will document the way they are used to do. Their documentation will be collected after the end of the study. Therapists in all groups will videotape one session of each child at four months of study-intervention. These tapes will be evaluated by trained raters, for procedural reliability and to describe regular care intervention.

Additional data on the working mechanisms will be collected by *in-depth interviews *with the families. Parents of children in the child-focused intervention approach and context-focused intervention approach group will be interviewed after the six months study-intervention period. Questions will be on parents experiences with the interventions, to get more insight into the advantages and disadvantages of the interventions. We expect that an 'informational redundancy'-effect will occur after interviewing half of the parents. To prevent that parents will be interviewed unnecessary, not all parents will be interviewed.

### Data analysis

Descriptive statistics including frequencies, means, standard deviations, and for nonparametric data medians and interquartile ranges will be calculated. Data will be analyzed based on an intention to treat-analysis. The effects of the three interventions will be evaluated using repeated-measures analysis of variance (group[3] × time[2]) with the outcome measures as the dependent variable. In case of a statistically significant interaction group × time effect, post-hoc analyses will be performed to determine between which groups the differences occur. To assess the effects during and after the six month study-intervention period, repeated-measures analysis of variance (group[3] × time[3]) with repeated contrasts will be used, with the same outcome measures.

To explore variables that might be related to the efficacy of the intervention, and that might explain possible variability between children, multiple regression analyses will be performed. The change scores on the PEDI-FSS will be the dependent variable, and the classification of gross motor abilities, classification of manual abilities, cognition of the child and coping style of the parents will be analyzed as independent variables.

The interviews with the parents on their experiences with the interventions, and therapists' preferences for intervention will be analyzed using qualitative analyses.

## Discussion

To provide the best intervention promoting outcome in children with developmental disabilities, it is necessary to examine which interventions are effective in randomized controlled trials and to study why a specific intervention is effective. Little is known about the efficacy and the working mechanisms of physical and occupational therapy interventions for children with CP. In this paper we have presented the background information, design, description of interventions and protocol for this study on the efficacy and working mechanisms of child-focused intervention approach and context-focused intervention approach compared to regular care intervention in mobility and self-care skills of children (2-3 years) with CP.

## Competing interests

The authors declare that they have no competing interests.

## Authors' contributions

MK and JG developed the original concept and wrote the grant application; all authors contributed to the design and the protocol of the study; MK, AK and OV are involved in the study implementation. EL is the program leader, and HR is the program coordinator of the research program LEARN 2 MOVE; MK is the project leader of LEARN 2 MOVE 2-3. All authors participated in the writing and or reviewing of this manuscript. All authors read and approved the final manuscript.

## Pre-publication history

The pre-publication history for this paper can be accessed here:

http://www.biomedcentral.com/1471-2431/10/80/prepub
